# Image-Based Hidden Damage Detection Method: Combining Stereo Digital Image Correlation and Finite Element Model Updating

**DOI:** 10.3390/s24154844

**Published:** 2024-07-25

**Authors:** Wei-Han Cheng, Hsin-Haou Huang

**Affiliations:** Department of Engineering Science and Ocean Engineering, National Taiwan University, Taipei 10617, Taiwan; r11525002@ntu.edu.tw

**Keywords:** stereo DIC, FEMU, damage assessment, non-contact method, model reconstruction

## Abstract

Maintenance and damage detection of structures are crucial for ensuring their safe usage and longevity. However, damage hidden beneath the surface can easily go unnoticed during inspection and assessment processes. This study proposes a detection method based on image techniques to detect and assess internal structural damage, breaking the limitation of traditional image methods that only analyze the structure’s surface. The proposed method combines full-field response on the structure’s surface with finite element model updating to reconstruct the structural model, using the reconstructed model to detect and assess hidden structural damage. Initially, numerical experiments are conducted to generate known damaged areas and parameter distributions. Data from these experiments are used to update the finite element model, establish and validate the proposed model updating method, and assess its accuracy in evaluating hidden damage, achieving an accuracy rate of 90%. Furthermore, discussions on more complex damage scenarios are carried out through numerical experiments to demonstrate the feasibility and applicability of the proposed method in reconstructing different forms of damage. Ultimately, this study utilizes stereoscopic digital imaging techniques to acquire full-field information on surfaces, and applies the proposed method to reconstruct the structure, enabling the detection and assessment of hidden damage with an accuracy rate of 86%.

## 1. Introduction

Structural health monitoring (SHM) is essential for evaluating the safety and reliability of structures. However, the limited number of sensors in SHM systems prevents direct monitoring of all structural components [1]. Therefore, finite element analysis has become an important tool to assist in SHM, as it provides global and local responses by establishing finite element models that match real-life conditions, including areas where sensors are not utilized. However, discrepancies often exist between numerical simulations and real-world results, where modeling errors in finite element models can lead to differences between the results obtained through numerical analysis and the actual situation. To effectively utilize numerical simulations for continuous monitoring of structures [2], damage detection [3,4], and predicting service life, reliability and accuracy are crucial [5,6]. This is where finite element model updating (FEMU) methods come into play. The primary purpose of FEMU methods is to calibrate and accurately reconstruct structural models based on the real experimental results from structural dynamic and/or static experiments, thereby reflecting the structure’s actual behavior [7].

In FEMU, the model updating problem is often reformulated as a structural optimization problem, where the objective is minimize errors between different types of numerical and experimental datasets, including structural dynamic responses [8,9], static data [10], or their combinations [11]. These datasets indicate the actual behavior of a structure: changes in the structure lead to variations in stiffness, which subsequently result in changes in structural characteristics. When utilizing structural dynamic responses for model updating, researchers not only consider mode shapes and natural frequencies but also incorporate Frequency Response Functions (FRF), Modal Elastic Residual Errors, and Modal Strain Energy (MSE). Besides utilizing structural dynamic responses for FEMU, model updating can also be performed using static data. In this scenario, the displacement and strain obtained from the static experiment are utilized for FEMU [10].

However, achieving precise structural models for damage detection through FEMU requires deploying a significant number of sensors to acquire experimental data [12]. If the experimental data are obtained from traditional sensors such as accelerometers, strain gauges, and fiber Bragg grating sensors, the high cost of obtaining experimental data directly limits the applicability of FEMU in various scenarios.

On the other hand, digital image correlation (DIC) has emerged as a powerful non-contact full-field detection method, having been proposed over the past 30 years [13]. Although 2D-DIC is a straightforward and efficient technique for detecting displacements and strain field within a plane, it frequently encounters perspective errors due to depth variations [14]. This occurs particularly when the surface of the object under examination is not flat or when imaging is not conducted with a vertical angle of incidence [15]. Therefore, in recent practical applications, 2D-DIC is often replaced by Stereo Digital Image Correlation (stereo DIC) systems composed of two or more cameras. In recent years, stereo DIC has been applied in various fields for full-field analysis, such as large-scale SHM [16], vibration measurement [17,18], and biomedical engineering [19]. In the field of large-scale SHM, unmanned aerial vehicles equipped with dual-camera systems have been used for on-site measurement and long-term monitoring of bridges, successfully detecting changes in bridge expansion joints and correlating them with seasonal variations, demonstrating the feasibility of using unmanned aerial vehicles equipped with stereo DIC systems for remote monitoring of large structures [16]. In vibration measurement, researchers have used high-speed cameras to establish stereo DIC systems for vibration measurement of rotating disks at high speeds and to reconstruct their modal shapes [18]. In biomedical engineering, a large set of multi-camera measurement devices has been established to create a 3D model of the human lower leg by surrounding the target with multiple cameras, enabling observation and measurement of the deformation of the entire surface of the lower leg. This demonstrates that stereo DIC, with its advantages of unrestricted three-dimensional digital imaging and the ability to detect larger target areas through the combination of multiple cameras, has considerable potential for development in various fields where sensor measurements are required [20]. The research results of the aforementioned teams regarding stereo DIC indicate that it serves as a non-contact detection method with the capability for local or full-field detection, while also possessing advantages such as simple setup and lower cost [21,22]. It has the potential to outperform traditional detection methods and thus holds significant promise for development in various fields requiring sensor-based measurements. In the field of damage detection, when structures experience damage, rapid changes in strain may occur in the affected areas. The capability of Digital Image Correlation (DIC) to analyze full-field displacement and strain fields makes it a valuable tool for damage detection [23]. However, a key limitation of optical methods such as DIC is their ability to analyze only the surface of structures. Therefore, in the literature, the focus of damage detection has often been on detecting cracks [24,25], leading to the development of several effective crack detection methods [26]. This study focuses on overcoming the limitation of optical methods in providing internal structural information.

Therefore, to address the limitations of traditional image methods, this study proposes a damage detection method based on image technology and FEMU, implemented under simple boundary conditions of uniaxial tension. This method utilizes Stereo DIC to capture the full-field responses of the structural surface as experimental data for calibrating the structural model. Through FEMU, a structural model closer to reality is reconstructed. Ultimately, this reconstructed model is employed for damage detection and assessment within the structure. Using the proposed method, internal information inaccessible to direct measurement by image techniques can be evaluated through finite element analysis. The reconstructed structural model effectively detects damage hidden beneath the surface, thereby achieving the goal of SHM.

The structure of article is as follows: In Section 2, we introduce the Stereo DIC method and model updating approach. Section 3 describes numerical experiments conducted with known numerical models to illustrate and validate the accuracy and feasibility of the proposed method. Section 4 contains a detailed discussion of the developed method, including uncertainties in initial parameter values and complex damage forms. Following that, Section 5 presents experiments conducted using Stereo DIC to obtain experimental data for model updating, providing experimental validation for the detection and assessment of actual hidden damage. Finally, Section 6 presents the conclusions of the research.

## 2. Methodology of the Image-Based Hidden Damage Detection Method

### 2.1. Framework Overview

**Figure 1:**
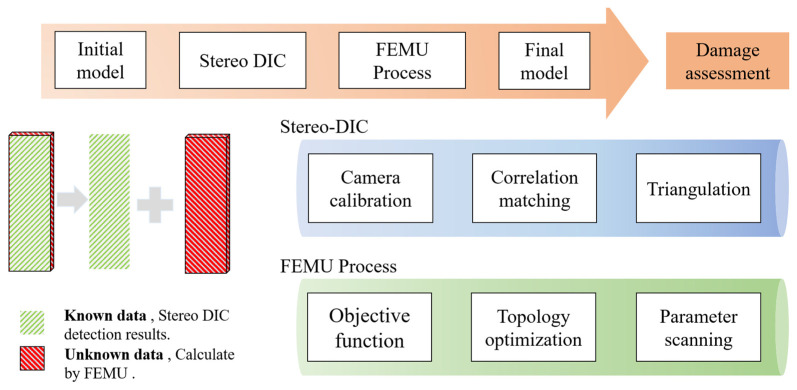
Overview of the Image-Based Hidden Damage Detection Method.

The proposed hidden damage assessment method, as illustrated in Figure 1, mainly consists of two approaches: (i) Stereo DIC and (ii) the FEMU method. This section presents the details of each method.

### 2.2. Stereo DIC

When using digital image correlation methods with a single camera, there are limitations, such as the requirement for the surface of the structure to be flat and deformation directions to remain parallel to the imaging plane of the camera; otherwise, out-of-plane errors may occur. However, not all structures are flat, and deformation directions may not necessarily be parallel to the imaging plane. To overcome these limitations and obtain more accurate detection results, this study establishes a Stereo DIC system through synchronous capture with dual cameras. Stereo DIC enables the analysis of three-dimensional displacement and strain of the surface of the test object, thus improving upon the shortcomings of traditional 2D-DIC, which can only measure in-plane variations.

The usage of Stereo DIC first requires camera calibration to establish the relationship between the cameras, such as their coordinates in world space. Then, using the matching algorithm of 2D-DIC, the positions of subsets at different times and viewpoints are obtained. By leveraging the correlation between the cameras and the corresponding positions of subsets in images from different viewpoints, stereoscopic reconstruction of subsets at different times is achieved through triangulation calculations. By computing subsets within the region of interest (ROI) at different times, reconstruction results of the structural surface within the at different times are obtained. Finally, by calculating the displacement and deformation of each point relative to its initial point, the temporal variation of the object’s displacement and deformation in space is obtained, thereby acquiring the displacement and strain field of the three-dimensional object surface over time. In the following content, the experimental and computational procedures of Stereo DIC will be introduced.

#### 2.2.1. Camera Calibration

The purpose of camera calibration is improve the accuracy and precision of camera imaging by reducing or eliminating various distortions that occur in camera imaging. This ensures that the camera’s imaging model better corresponds to the real-world model, thereby enhancing the quality of photographs and the accuracy of measurements. Camera calibration allows obtaining the camera’s pose, which can be used to determine its position and orientation during the imaging process. Through camera pose estimation, both the intrinsic and extrinsic parameter matrix can be determined, thereby achieving transformation between the camera system and world system. This is crucial for precise three-dimensional measurements and reconstructions, with Zhang’s calibration method being widely used in current camera calibration techniques [27].

Zhang’s calibration method utilizes a checkerboard calibration board for camera calibration. After obtaining images of the calibration board, automated corner detection can be used to obtain the position of checkerboard. During calibration, the world coordinate system is fixed on the checkerboard, with any point on the calibration board having Z = 0. Since the specifications of the checkerboard are known, world coordinates on the calibration board can be precisely determined. By matching the pixel coordinates of the checkerboard with their corresponding physical coordinates, the camera’s intrinsic and extrinsic parameters can be computed. These parameters describe the camera’s imaging model, thereby achieving calibration of the camera imaging.

#### 2.2.2. Correlation Matching

DIC is a non-contact technique for full-field measurement that integrates optical imaging analysis. It involves recording images of the test object using digital cameras to capture its displacement and deformation. By processing the captured images using computer algorithms, DIC can provide comprehensive information such as the displacement and strain of every tracking point on the surface. In image analysis and tracking, it is impractical to track individual pixels directly. Hence, during tracking, subsets are extracted around desired tracking points as the tracking unit, and a ROI is defined as the search area. In the computation of full-field DIC, an initial image is first set, usually chosen as the image recorded in the initial state. Then, an ROI is selected from the image, and divided into numerous subsets for sequential matching. Through DIC algorithms, the similarity between the pixels of the initial subset and those of the deformed images is analyzed, providing full-field displacement information, including translations, rotations, and deformations. To further enhance accuracy, sub-pixel algorithms can be utilized to obtain sub-pixel resolution of displacement, enabling high-precision tracking and revealing surface deformations imperceptible to the naked eye. In the following sections, the computational workflow of DIC will be outlined.

When conducting image analysis using DIC, the subset from the initial image serves as the reference subset, while the subsets from subsequent deformed images used for matching are referred to as search subsets. To quantify the degree of similarity during similarity matching, DIC employs the correlation coefficient to define the similarity between subsets. In this study, for estimating whole-pixel displacements, the Normalized Cross Correlation (NCC) method is utilized [28], as shown in (1), to compute the correlation coefficient field between subsets and images. By calculating the maximum correlation coefficient value, the position of the reference subset in the deformed image can be determined. Comparing the changes in subset positions before and after deformation allows for the calculation of whole-pixel displacement.

(1) $$C_{Ncc}=\frac{\sum_{\left(i,j\right)\in S}\left(f\left({\tilde{x}}_{ref_{i}},{\tilde{y}}_{ref_{j}}\right)-\bar{f}\right)\left(g\left({\tilde{x}}_{cur_{i}},{\tilde{y}}_{cur_{j}}\right)-\bar{g}\right)}{\sqrt{\sum_{\left(i,j\right)\in S}{\left[f\left({\tilde{x}}_{ref_{i}},{\tilde{y}}_{ref_{j}}\right)-\bar{f}\right]}^{2}{\left[g\left({\tilde{x}}_{cur_{i}},{\tilde{y}}_{cur_{j}}\right)-\bar{g}\right]}^{2}}}$$

*S* represents the total pixels in the subset, f(x,y) represents reference subset, and  g(x,y) represents search subset, while $\bar{f}$ and $\bar{g\;}$ represent the mean grayscale intensity of reference and deformed subset.

When using DIC to inspect the surface of a structure, subsets within the feature regions of the surface may undergo deformation due to external forces such as stretching and shearing, leading to changes in the shape of the search subset in subsequent images. Common deformations include rigid body rotation, first-order deformation, and second-order deformation. In addition to the limitations of whole-pixel calculations in terms of accuracy, there can be significant errors in the correlation coefficient when dealing with such deformations of the search subset. To reduce the impact of search subset deformation on the calculation results, sub-pixel algorithms compute a deformation function (warp function) for subsets within the feature regions and use Inverse Compositional Gauss-Newton method (IC-GN) to calculate the deformation function of the subset [29].

In the numerical computation process of the IC-GN, the subset extracted from the reference image is referred to as the reference subset *f*, and the subset extracted from the deformed image is referred to as the search subset *g*. In the initial calculation of the algorithm, the whole-pixel displacement solution for the reference and search subsets is obtained using NCC. This solution is then used as the initial guess for the deformation parameter P of the subset, as shown in (2), allowing the subset to deform accordingly.

(2) $$P=\left[\begin{array}{ccc} \begin{array}{ccc} \begin{array}{cc} u & v \end{array} & \frac{du}{dx} & \frac{du}{dy} \end{array} & \frac{dv}{dx} & \frac{dv}{dy} \end{array}\right]$$

During the sub-pixel displacement estimation stage, for computational convenience, we employ the Zero-Normalized Sum of Squared Differences (ZNSSD) as the criterion for comparing correlation coefficients, as shown in (3):

(3) $$C_{ZNSSD}={\sum_{i=1}^{I}\left[\frac{f(W(x+\Delta x,y+\Delta y,0))-\bar{f}}{\Delta f}-\frac{g(W(x+\Delta x,y+\Delta y,P))-\bar{g}}{\Delta g}\right]}^{2}$$

where W(x+Δx,y+Δy,P) represents the first-order deformation function, while Δf and Δg represent the standard deviation of reference and deformed subset.

Setting its partial derivative with respect to ΔP to zero yields the following Equation (4):

(4) $$\frac{\partial C_{ZNSSD}}{\partial \Delta P}=0=\sum_{i=1}^{I}\frac{2}{\Delta f}{\left[\nabla f\frac{\partial W}{\partial P}\right]}^{T}\left[\frac{f-\bar{f}}{\Delta f}+\frac{1}{\Delta f}\frac{df}{dW}\frac{\partial W}{\partial P}\Delta P-\frac{g-\bar{g}}{\Delta g}\right]$$

In every iteration, the equation is solved using the least squares method, and the deformation parameter P will be updated using ΔP. In this study, the stopping criterion for iteration is when ΔP is less than 1 × 10^−6^ and the iteration exceeds 100 times.

#### 2.2.3. Triangulation

**Figure 2:**
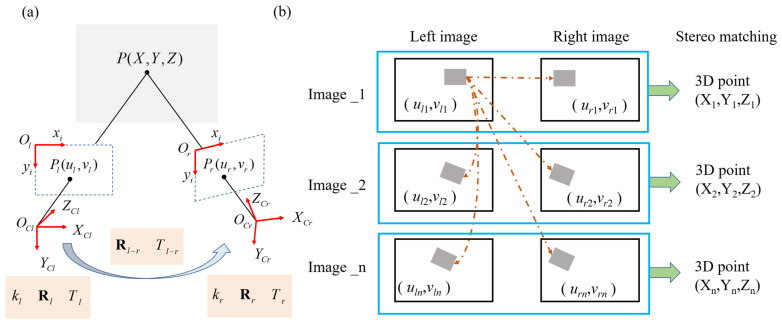
Stereo DIC schematic: (**a**) Triangulation; (**b**) stereo reconstruction on each image pair at different times.

After completing camera calibration, we can obtain the intrinsic and extrinsic parameters of each camera. With these parameters, we can establish a stereo camera system to obtain depth information. Through triangulation using camera parameters and similarity matching results, we can obtain the corresponding spatial coordinates, as depicted in Figure 2a. Next, we will introduce the process of solving for the true three-dimensional spatial coordinates *X*, *Y*, and *Z* through stereo camera triangulation.

In a stereo camera system, images are captured from two different cameras, each with its own pinhole imaging model. Suppose there is a known point P(*X*,*Y*,*Z*) in space, and this point corresponds to a pixel in both images, denoted as *P*_*l*_(*u*_*l*_,*v*_*l*_) and *P*_*r*_(*u*_*r*_,*v*_*r*_) respectively. Through this correspondence, we can formulate the pinhole imaging model for the corresponding points in both cameras.

With the external parameter matrices of the two cameras, we can establish the correspondence between the two cameras in space. This relationship corresponds to the rotation matrix and translation matrix, denoted as $R_{l-r}$ and $T_{l-r}$ respectively. The models of the two cameras can be simplified to Equations (5) and (6):

(5) $$s\left[P_{l}\right]=\left[k_{l}\right]\left[P\right]$$

(6) $$s\left[P_{r}\right]=\left[k_{r}\right](\left[R_{l-r}\right]\left[P\right]+T_{l-r})$$

where $k_{r}$ and $k_{l}$ are the intrinsic parameter matrices of the right and left cameras.

Expanding and solving these equations:

(7) $$f_{xl}X+\left(c_{xl}-u_{l}\right)Z=0$$

(8) $$f_{yl}Y+\left(c_{yl}-v_{l}\right)Z=0$$

(9) $$\begin{array}{c} {}[(u_{r}-c_{xr})R_{31}-f_{xr}R_{11}]X+[(u_{r}-c_{xr})R_{32}-f_{xr}R_{12}]Y\\ \;\;\;\;\;\;\;\;\;\;\;\;\;\;\;\;\;\;+[(u_{r}-c_{xr})R_{33}-f_{xr}R_{13}]Z=f_{xr}T_{1}-\left(\left(u_{r}-c_{xr}\right)\right)T_{3} \end{array}$$

(10) $$\begin{array}{c} {}[(v_{r}-c_{yr})R_{31}-f_{yr}R_{11}]X+[(v_{r}-c_{yr})R_{32}-f_{yr}R_{12}]Y\\ \;\;\;\;\;\;\;\;\;\;\begin{array}{cc} & + \end{array}[(v_{r}-c_{yr})R_{33}-f_{yr}R_{13}]Z=f_{yr}T_{1}-\left(\left(v_{r}-c_{yr}\right)\right)T_{3} \end{array}$$

The camera parameters in this study have been obtained through Zhang’s camera calibration method, resulting in the parameters for each camera. By substituting these known parameters into Equations (7)–(10), the three-dimensional spatial coordinates *X*, *Y*, and *Z* of point P can be solved.

To calculate structures’ displacement and deformation, Stereo DIC not only performs spatial matching through stereo reconstruction but also incorporates temporal matching for calculation. In practice, subsets within the ROI of the pre-selected reference images are matched not only across different viewpoints but also across different time frames. As shown in Figure 2b, this process provides the positions of all subsets in the images at different times and viewpoints. Then, by performing stereo reconstruction on each pair of images at different times and viewpoints, the three-dimensional coordinates of all points at different times can be obtained, enabling the reconstruction of the surface in three dimensions over time. Therefore, by examining the coordinate changes of the same point at different times, displacement information can be calculated, as depicted in Figure 2b.

### 2.3. FEMU Method

#### 2.3.1. FEMU Problem Definition

In this research, FEMU is defined as structural optimization by setting up an objective function. Through this approach, the finite element model is updated to minimize the difference between the finite element analysis results and the experimental measurements. The proposed FEMU process is divided into two parts. Firstly, the model is updated using a topology optimization method to obtain the initial distribution of structural stiffness parameters. To further improve the accuracy of parameter updating, a small parameter scan is conducted based on the parameter distribution obtained from the topology optimization.

Objective function is defined as the error between the experimental data and the finite element analysis data, as shown in (11):

(11) $$c={\left(\frac{D^{\exp}-D^{femu}(x)}{D^{\exp}}\right)}^{2}\times 100\%$$

where $D^{\exp}$ represents the experimental data, which may include $\varepsilon_{xx}^{exp},\varepsilon_{yy}^{exp},{\;\gamma }_{xy}^{exp},u^{exp}$ and $v^{exp}$. $D^{femu}$ represents the finite element analysis data during the FEMU process, which similarly may include finite element analysis data such as $\varepsilon_{xx}^{femu},\varepsilon_{yy}^{femu},{\;\gamma }_{xy}^{femu},u^{femu}$ and $v^{femu}.$

#### 2.3.2. Topology Optimization

This study employs the density method within topology optimization for calculations. This method discretizes the design domain and introduces a variable in each element to represent the importance of material within that region, considering given loads, boundary conditions, and constraints. The study utilizes the simplest and most widely used penalization methods in density-based optimization called the Solid Isotropic Material with Penalization (SIMP) method. In this method, a variable is defined using an initial Young’s modulus $E_{0}$ and a relative density coefficient, referred to as stiffness density $x_{e}$ in this study. Young’s modulus $E_{e}$ for each element is defined using a power law, as shown in (12):

(12) $$E_{e}=E_{e}(x_{e})=x_{e}^{\rho }E_{0},\;x_{e}\in [0,\;1]$$

where ρ is the penalty factor. Previous literature suggests that a value of 3 for ρ yields better results [30,31]. In traditional SIMP methods, when the stiffness density $x_{e}$ approaches 0, it leads to singularity in the element stiffness matrix. Therefore, it is defined as (13) [32]:

(13) $$E_{e}=E_{e}(x_{e})=E_{\min}+x_{e}^{\rho }(E_{0}-E_{\min}),\;x_{e}\in [0,\;1]$$

where E_0_ >> E_min_.

Moreover, to prevent the formation of checkerboard patterns in the optimization results and ensure the existence of solutions to the topology optimization problem, a density filter is applied to the analysis of stiffness density distribution:

(14) $${\tilde{x}}_{e}=\frac{1}{\sum_{i\in N_{e}}H_{ei}V_{i}}\sum_{i\in N_{e}}H_{ei}V_{i}x_{e}$$

(15) $$N_{e}=\left\{i:\mathrm{dist}(e,i)⩽R_{min}\right\}$$

where $N_{e}$ is elements whose distance dist(e,i) is less than the radius of the filter, $V_{i}$ represents the volume of each element, which is defined as unity in this calculation method, and $H_{ei}$ is the weight factor, defined as (16):

(16) $$H_{ei}=R_{min}-\mathrm{dist}(e,i)$$

After density filtering, SIMP can be rewritten as (17):

(17) $$E_{e}({\tilde{x}}_{e})=E_{min}+{\tilde{x}}_{e}^{\rho }(E_{0}-E_{min}),{\tilde{x}}_{e}\in [0,\;1]$$

Next, to estimate the stiffness density distribution, the objective function needs to be analyzed through sensitivity analysis. Sensitivity analysis helps determine how changes in stiffness density affect the objective function, thereby facilitating stiffness density calculations in topology optimization. Since the stiffness density calculation results undergo filtering through the density filter, this process needs to be considered in the sensitivity analysis calculations as well. The integrated sensitivity analysis calculation method for each element is given by:

(18) $$\begin{array}{c} \frac{\partial c_{e}({\tilde{x}}_{e})}{\partial x_{e}}=\frac{\partial c_{e}({\tilde{x}}_{e})}{\partial {\tilde{x}}_{e}}\frac{\partial {\tilde{x}}_{e}}{\partial x_{e}} \end{array}$$

After obtaining the results of sensitivity analysis, this study employs Optimality Criteria (OC) for the parameter optimization method in the topology optimization process. The OC method selects the updated parameter values based on the optimality criteria set within the optimization method. In this study, the OC method is configured based on the constraint $x_{e}$ ∈ [0, 1] as shown in (19) and (20). In this method, the optimal adaptation condition is expressed as $B_{e}=1$. To satisfy the constraint conditions in each calculation process, the Lagrange multiplier λ is introduced to estimate the optimal adaptation. Based on the definition of volume as unit volume, Equation (21) is derived. Therefore, by solving for the Lagrange multiplier λ, the updated parameter x_e_^new^ can be obtained. In numerical computation, λ is obtained using the Bisection method.

(19) $$x_{e}^{\mathrm{new}}=\left\{\begin{array}{cc} \max(0,x_{e}-m) & \mathrm{if}\;x_{e}B_{e}\leq \max(0,x_{e}-m)\\ \min(1,x_{e}+m) & \mathrm{if}\;x_{e}B_{e}\geq \min(1,x_{e}+m)\\ x_{e}B_{e} & otherwise \end{array}\right.$$

(20) $$B_{e}=\frac{-\frac{\partial c_{e}}{\partial x_{e}}}{\lambda \frac{\partial V_{e}}{\partial x_{e}}}$$

(21) $$\frac{\partial V_{e}}{\partial x_{e}}=1$$

where m is the parameter adjustment limit, used to prevent too large changes from occurring at once [33]. Finally, the computation stops when the maximum iteration count reaches 200 or when the termination criterion (22) is satisfied:

(22) $$\max(|{x_{e}}^{\mathrm{new}}-x_{e}|)⩽0.01$$

#### 2.3.3. Parameter Scanning

After completing model updating using the topology optimization algorithm, the parameter distribution obtained from the topology optimization algorithm can be obtained. To improve the accuracy of parameter estimation for structurally damaged areas, a second-stage model updating method called parameter scanning is introduced. In this stage, to determine the damaged areas, half of the initial stiffness is used as a threshold. Regions with stiffness greater than this value are defined as healthy areas, while regions with stiffness less than this value are defined as damaged areas. In the parameter scanning process, E_H_ is selected in a decreasing manner based on stiffness density with the same density intervals, selecting an array of parameters N_P_. E_D_ is selected in a similar way, also with N_P_ parameter combinations. A total of N_P_^2^ computations are performed, and the final calculation result is the error computed based on the objective function. Finally, the parameter combination with the smallest error within the N_P_^2^ combinations is selected as the final result of the model parameter update.

## 3. Establishment and Validation of Model Updating Methods

### 3.1. Initial Setup of Model Updating

#### 3.1.1. Initial FEM Model

**Figure 3:**
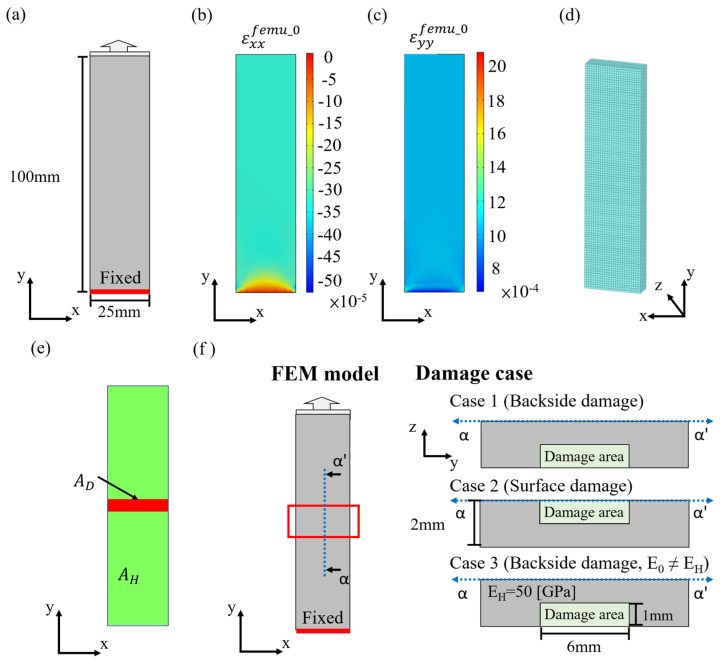
Initial setup of model updating: (**a**) Initial finite element model; strain field (**b**) $\varepsilon_{xx}^{femu\_0}$, (**c**) $\varepsilon_{yy}^{femu\_0}$; (**d**) topology optimization model; (**e**) estimate healthy area A_H_ and damaged area A_D_; (**f**) numerical experimental test dataset.

To reconstruct the model, an initial FEM model must first be created, with boundary conditions set to match the actual situation for numerical simulation. In this study, the discussion on structural model reconstruction is based on the scenario of a uniaxial tensile test. Therefore, the finite element simulation is set up for tensile conditions, as shown in Figure 3a, with the specimen dimensions set to 25 mm × 100 mm × 2 mm. Finite element analysis results are shown in Figure 3b,c.

#### 3.1.2. Topology Optimization Model

To estimate the distribution of model parameters using topology optimization, an optimization model of the same scale as the initial finite element model must be established for computation. According to the specimen dimensions, the topology optimization model is constructed with 25 × 100 × 8 optimization elements, as shown in Figure 3d. In the topology optimization calculation, an initial overall density value must be assigned, which gives each optimization element the same initial stiffness density coefficient x_e_. In this study, the percentage error is calculated by comparing experimental data with initial finite element analysis data, as shown in Equation (11). Subsequently, the Otsu method is applied for binarization to preliminarily estimate damage regions on the surface, obtaining the possible healthy area A_H_ and damaged area A_D_, as illustrated in Figure 3e.

In the topology optimization, the initial stiffness density coefficient x_e_ is determined through a weighted average of the healthy area A_H_ and the damaged area A_D_, as shown in Equation (23). Here, $x_{e}^{H}$ and $x_{e}^{D}$ are the weights for the healthy and damaged areas, respectively. Since $x_{e}^{H}\;$ corresponds to the healthy area, it is defined as 1. The value of $x_{e}^{D}$ is defined based on the indicator E_lim_ used for determining the healthy and damaged regions of the structure, as shown in (24).

(23) $$x_{e}=\left(\frac{A_{H}\times x_{e}^{H}+A_{D}\times x_{e}^{D}}{A_{H}+A_{D}}\right)$$

(24) $$E_{lim}=\phi_{E}\times E_{0}$$

ϕ_E_ is the threshold value set for the decrease in Young’s modulus. This value limits the allowable reduction in strength and is used to distinguish the damaged area. ϕ_E_ is set to 0.5 [34].

Based on the relationship between Young’s modulus E_e_ and the stiffness density x_e_, along with the damage determination criterion (24), the stiffness density value corresponding to the damaged area $A^{D}$ can be estimated. This value is then used as the weight $x_{e}^{D}$ for the damaged area, as shown in Equation (25). This process ultimately yields the initial stiffness density coefficient x_e_ as defined in this study Equation (26).

(25) $$x_{e}^{D}={\left(\phi_{E}\right)}^{\frac{1}{\rho }}$$

(26) $$x_{e}=\left(\frac{A_{H}\times 1+A_{D}{\left(\phi_{E}\right)}^{\frac{1}{\rho }}}{A_{H}+A_{D}}\right)$$

#### 3.1.3. Numerical Experimental Dataset

To validate whether the FEMU method established in this study can accurately reconstruct the distribution of structural parameters to achieve damage localization and assessment for hidden damages, numerical experimental test data are generated through finite element analysis. Three types of damage scenarios are investigated, including damage located on the backside, damage located on the front side, and damage located on the backside with initial material parameter errors, as shown in Figure 3f. After conducting numerical analysis on the configured damage models, surface data of the structure are obtained, which serve as the numerical experimental test dataset. The predefined material parameter distribution from the numerical experiments serves as the ground truth. By comparing the parameter distribution of the updated model with the actual parameter distribution, proposed model updating method accuracy can be analyzed.

The objectives of this section are as follows:

1. To simulate and obtain experimental datasets with known parameter distributions through numerical experiments, using the model updating method established in this study for model reconstruction. In this process, the parameters for the healthy region and the damaged region are defined as E_H_ and E_D_ and summarized in Table 1.
2. After model reconstruction using the model updating method established in this research, the parameter distribution of the reconstructed model can be obtained. By comparing it with the parameter distribution of the simulated dataset from numerical experiments, the effectiveness of model reconstruction can be evaluated.

### 3.2. Topology Optimization Parameter Distribution Estimation

#### 3.2.1. Topology Optimization Model Updating

Obtaining the results of numerical experimental analysis, a model updating process is performed using topology optimization. In the computation process, the initial model is compared with the experimental data of Case 1, and its sensitivity is calculated. Based on sensitivity analysis results, the distribution of model parameters to be used in the next finite element analysis calculation is determined. Subsequently, finite element analysis of the updated model yields the displacement and strain field distributions after parameter updating under the same conditions.

**Figure 4:**
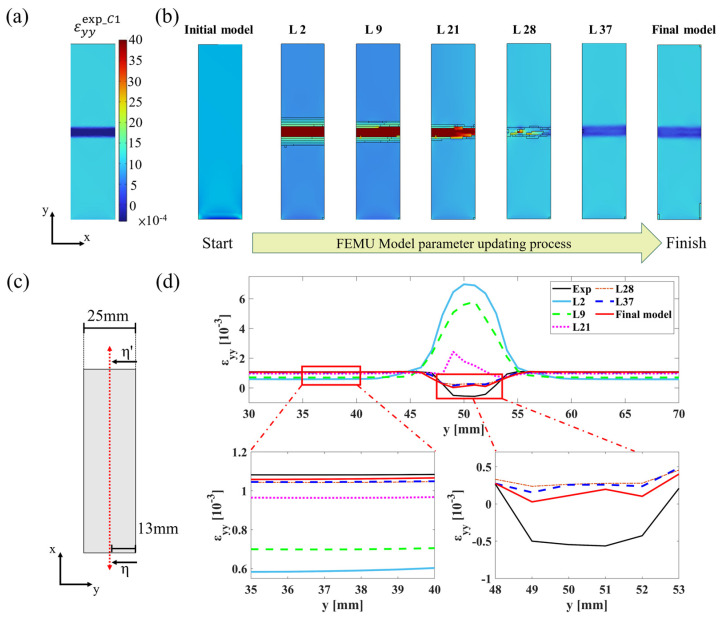
Result of topology optimization model updating: (**a**) case 1 FEA result; (**b**) topology FEMU process strain field; (**c**) surface profile η−η’ for data extracted; (**d**) strain field profile η−η.

To visually demonstrate the changes during the FEMU process, the strain field $\varepsilon_{yy}^{femu}$ is used to represent the changes in process, as in Figure 4a,b, where L# denotes the computation and update iterations. Additionally, data are extracted from the surface profile η−η′, as shown in Figure 4c, to illustrate the numerical changes in the $\varepsilon_{yy}^{femu}$ values along this profile, as depicted in Figure 4d.

**Figure 5:**
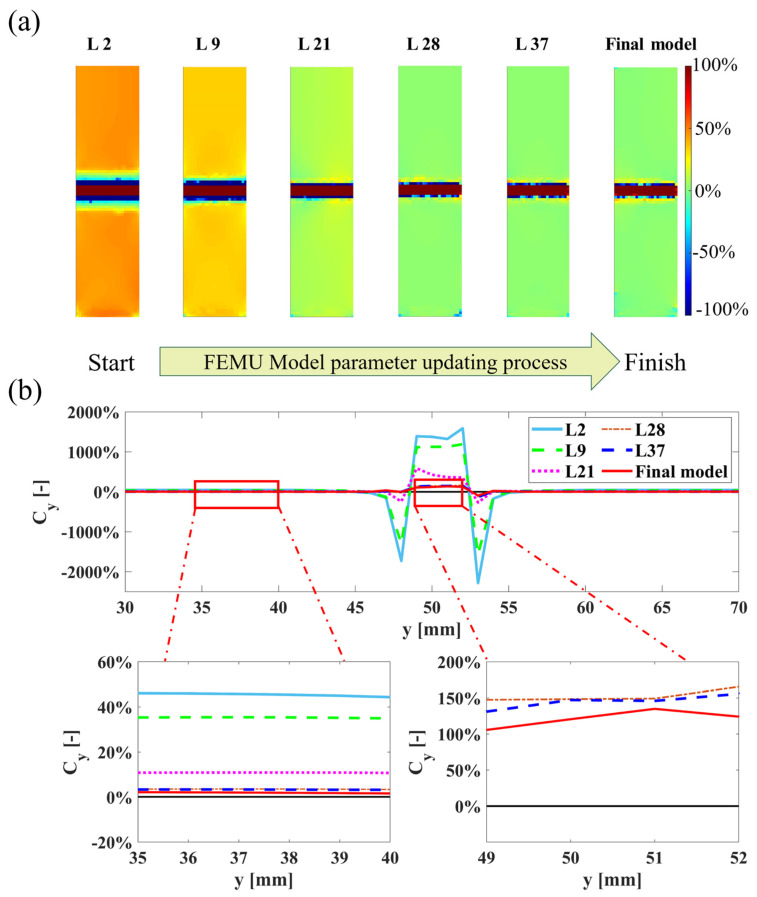
Topology FEMU process strain error; (**a**) strain error field; (**b**) error profile η−η’.

Furthermore, to better illustrate the variation in errors during the process, the error between strain field $\varepsilon_{yy}^{femu}$ and numerical experimental data is calculated, as shown in Equation (27). This process intuitively demonstrates that as the model updating progresses, the model approaches the numerical experimental analysis results, as depicted in Figure 5a. Similarly, the surface data along the selected profile are used to visualize the errors, providing a clearer view of the changes in error distribution Figure 5b.

(27) $$C_{y}=\frac{\varepsilon_{yy}^{\exp}-\varepsilon_{yy}^{femu}}{\varepsilon_{yy}^{\exp}}\times 100\%$$

#### 3.2.2. Model Updating Accuracy Analysis

**Figure 6:**
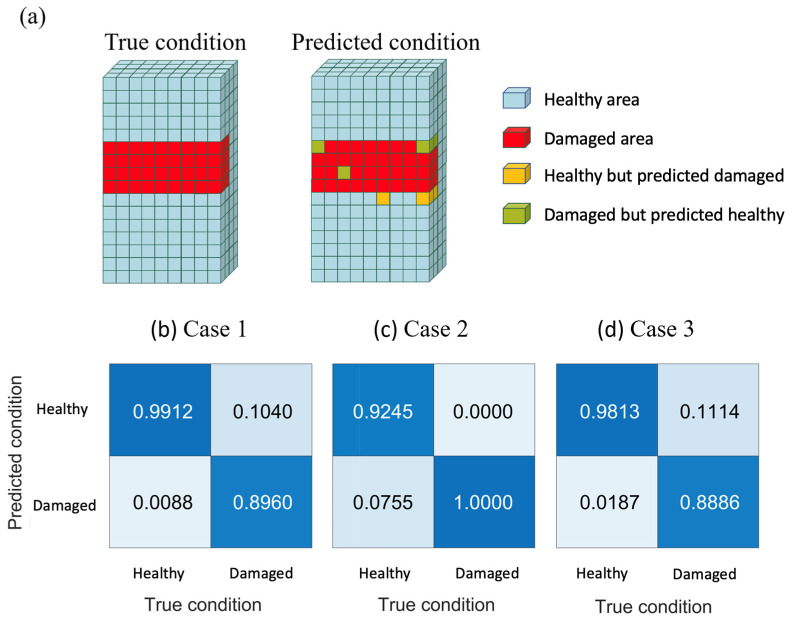
Accuracy analysis and parameter scanning results: (**a**) Accuracy analysis schematic; results of accuracy analysis (**b**) Case 1, (**c**) Case 2 and (**d**) Case 3.

To assess the accuracy of the model updating method proposed in this research for reconstructing damage models, this section utilizes the numerical experimental test data as the parameter distribution of the true model. The model calculated through topology optimization parameter distribution estimation is defined as the predicted model. In the predicted model, the regions of structural strength reduction, indicating the location of defects, can be accurately and clearly identified. At this point, using the predefined criterion value E_lim_, the predicted model is divided into the healthy area $A_{H}^{top}$ and the damaged area $A_{D}^{top}$, as shown in Equation (28). When comparing the predicted model with the true model, four possibilities can be observed: correctly predicting the healthy area, correctly predicting the damaged area, incorrectly predicting the healthy area as damaged, and incorrectly predicting the damaged area as healthy, as Figure 6a. By extracting these elements and calculating their respective percentages, the accuracy of damage model reconstruction in this study and its corresponding relationships can be determined.

(28) $$\left\{\begin{array}{c} \begin{array}{cc} A_{H}^{top} & if\;E_{top}>E_{lim} \end{array}\\ \begin{array}{cc} A_{D}^{top} & if\;E_{top}<=E_{lim} \end{array} \end{array}\right.$$

From Figure 6b,d, it can be observed that in the accuracy analysis of damage reconstruction in numerical experiments, each group exhibited good accuracy in estimating both the healthy and damaged areas. For instance, in the case of back injury in Case 1, approximately 99% of the healthy area was successfully predicted, while around 90% of the damaged area was correctly predicted. Additionally, it is noteworthy that the only difference between Case 3 and Case 1 lies in the parameter value settings, and such differences only resulted in a slight decrease in the reconstruction accuracy.

### 3.3. Final Parameter Update via Parameter Scanning

#### Parameter Scanning Model Updating

After updating the structural model through topology optimization, it was found that the results showed good accuracy in reconstructing and identifying the damaged areas. However, there was a significant error in the evaluation of parameter values. To improve the accuracy of parameter estimation in model updating, parameter scanning will be conducted for both the damaged and healthy areas.

**Figure 7:**
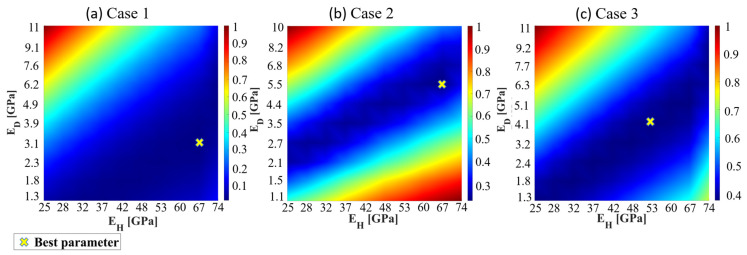
Results of parameter scanning: (**a**) Case 1, (**b**) Case 2, and (**c**) Case 3.

Based on the model estimated by topology optimization, the structure is divided into healthy and damaged areas. Several sets of parameters are established for parameter scanning. Finite element analysis is then performed for all combinations to obtain corresponding analysis results. The error is calculated using an objective function, and all results are normalized by defining the maximum value as 1, as shown in Equation (29). A heat map is generated from the normalized data Ps, as illustrated in Figure 7a,c. The parameter combination with the smallest error, indicated by the red dot in Figure 7a,c, is selected as the final parameter set for model reconstruction. This point represents the optimal solution obtained through parameter scanning.

(29) $$P_{s}=\frac{c}{c_{max}}$$

where c is the error calculated using the objective function, and $c_{max}$ is the maximum error obtained during parameter scanning.

(30) $$Error=\frac{Default\;parameters-Final\;parameter}{Default\;parameters}$$

The optimal parameter combination obtained through parameter scanning is summarized in Table 2. Using (30), the error between the updated and the preset parameters is calculated. The table demonstrates that parameter scanning effectively enhances the parameter estimation accuracy in the damaged areas.

**Figure 8:**
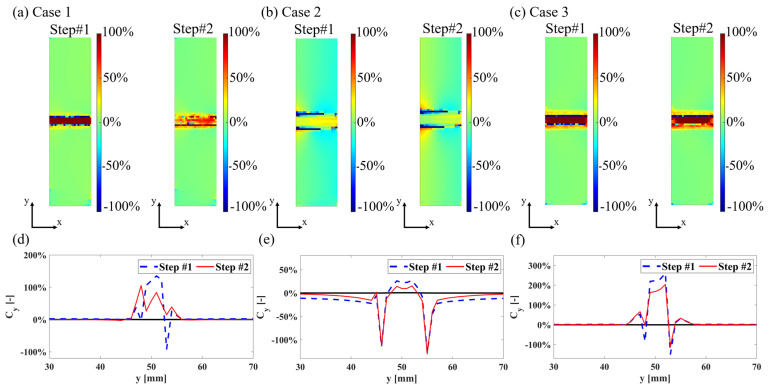
Results of error analysis: surface error distribution: (**a**) Case 1, (**b**) Case 2, and (**c**) Case 3; surface error profile η−η’ (**d**) Case 1, (**e**) Case 2, and (**f**) Case 3.

An error analysis is then performed again by comparing the surface error distribution before and after parameter updating, as shown in Figure 8a,c. It can be clearly seen that parameter scanning significantly reduces the surface error in the numerical analysis results. Additionally, the error variation along the selected profile is extracted, and the improvement in the accuracy of the numerical model analysis due to parameter updating is directly evident from the numerical data, as shown in Figure 8d,f.

**Figure 9:**
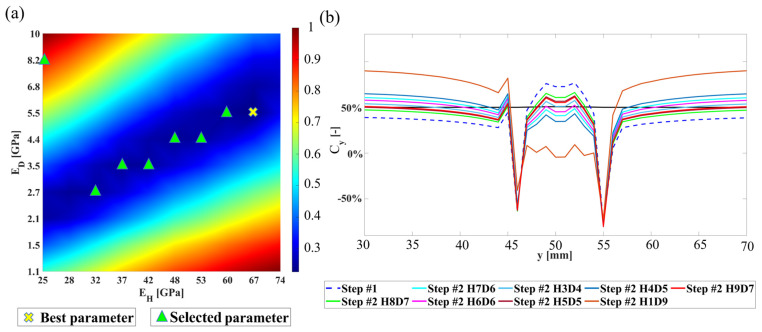
Parameter scanning discussion: (**a**) data selection; (**b**) surface error profile η−η’.

During the parameter scanning analysis in this phase, a noteworthy phenomenon was observed and discussed. When presenting the results of parameter scanning as heat maps, it was noted that the central region exhibited smaller errors, closely approaching the minimum value. To understand the differences between these values, which are generally smaller and close to the minimum, several values were selected for further examination, as shown in Figure 9a. Analysis of these points revealed that the overall error regions were similar, indicating that certain areas exhibited smaller errors under different parameter combinations, while other areas showed larger errors. Therefore, selecting the parameter combination with the smallest total error can minimize the difference between the analysis results and experimental results, as shown in Figure 9b, where H#D# represents the group corresponding to the parameter scan, and specific parameter values can be referred to by the green dots in Figure 9a.

## 4. Numerical Experiment Discussion on Model Updating for Damage Detection

### 4.1. Discussion on Initial Material Parameter Errors

In the process of establishing the model updating workflow, it is essential to create an FEM model with initial material parameters in finite element analysis software. As discussed previously in Case 3, deliberate parameter errors were introduced for discussion. In the analysis results of Case 3, this study found that under the proposed updating framework, deviations in initial parameter settings have a slight impact on the reconstruction results of both damaged and healthy regions. To further understand the influence of initial material parameter errors on the reconstruction results, several other cases with different degrees of parameter errors were tested under the same damage scenario as Case 3. This aimed to explore the range of errors introduced in structural reconstruction when initial parameters are inaccurately set. Table 3 summarizes the parameters used in these numerical experiments, where E_H_ represents the material parameters used in numerical experiments, and E_0_ represents the initial material parameters for FEMU.

**Figure 10:**
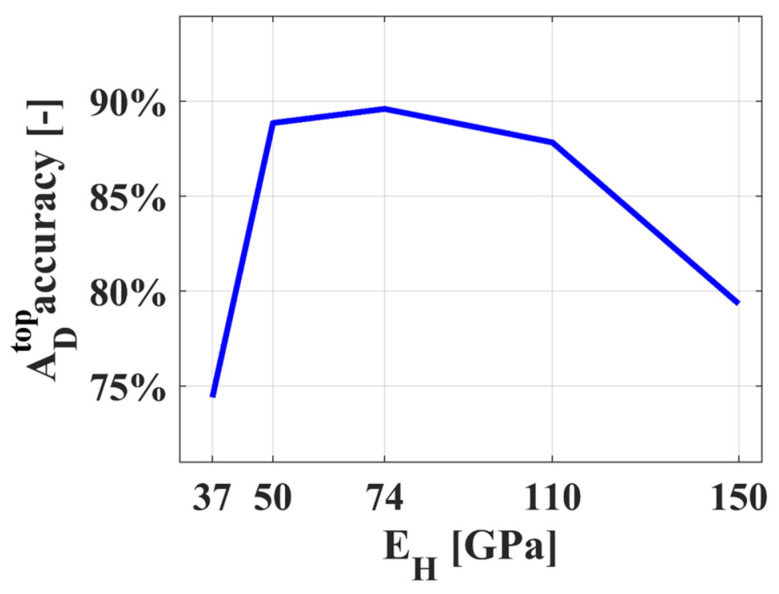
Accuracy of damaged area reconstruction under different parameters.

The focus is discussing the accuracy of reconstructing the damaged area $A_{D}^{top}$. The numerical experiment models are set as the true models, while the models reconstructed through the model updating process are considered as predicted models. By comparing the true damaged area settings with the reconstructed damaged areas in the predicted models, the accuracy of the predicted models in capturing the damaged area can be examined. The results are shown in Figure 10.

From the analysis results, it is observed that when there is a certain degree of error in the initial parameters, the difference in the reconstruction results of the damaged area is not significant compared to when the initial parameters are accurate. However, as the error in the initial parameters increases, the error in model reconstruction also increases. Therefore, while a certain level of error in the finite element analysis model setup may be acceptable, this error should not be too large. In practical applications, it is necessary to evaluate the initial structure parameters to avoid the influence of parameter setting errors on the analysis results.

### 4.2. Discussion on Numerical Experiment Damage Cases

#### 4.2.1. Experimental Objectives and Numerical Experiment Case Setup

**Figure 11:**
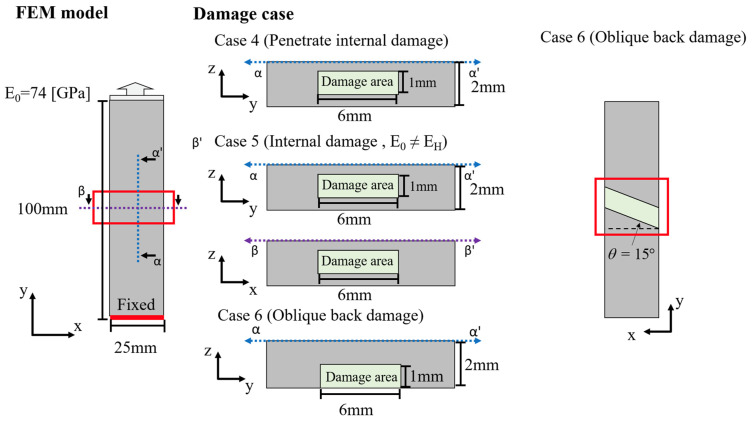
Numerical experiment damage discussion cases setup.

While this study illustrates and validates its proposed model updating process using simple numerical cases, real-world structural damages are often more diverse. In order to examine the effectiveness of the FEMU process established in this study under different damage scenarios, this section will discuss damage cases that are different from those discussed in previous sections. These new damage cases include two types of internal damages and oblique damage to the rear, as shown in Figure 11. Numerical experiments will be conducted with the aforementioned damage forms, taking into account deviations in initial values to reflect real-world conditions. Additionally, the model parameter E_H_ will also undergo slight changes and may not be identical to the model updating parameter E_0_, as listed in Table 4. Through numerical experiment results and the subsequent model updating process, structural reconstruction results will be obtained. These results will facilitate damage detection and assessment, allowing for a discussion on the effectiveness of model reconstruction under different damage forms.

#### 4.2.2. Results and Discussion

**Figure 12:**
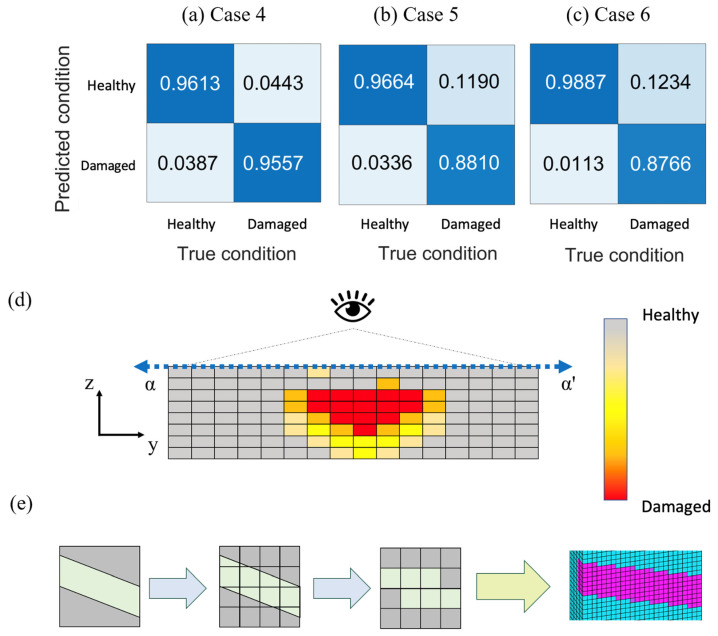
Numerical experiment damage discussion cases—setup and results: damage discussion cases setup; results of accuracy analysis: (**a**) Case 4, (**b**) Case 5 and (**c**) Case 6; (**d**) reconstruction of internal damage; (**e**) reconstruction of oblique back damage.

The FEMU process proposed in this research allows for the acquisition of structural reconstruction results and parameter estimation results. An analysis of the accuracy of structural reconstruction reveals that satisfactory estimates of the damaged areas can be obtained for different damage types, as shown in Figure 12a,c. It is worth noting that in the reconstruction of internal damage, compared to cases where damage is purely located on the front or back surfaces, the results appear somewhat imperfect. The surface and back regions are prone to being misjudged as damaged. Nevertheless, in the model calculation results, it can be observed that the central internal region is the primary damaged area, as shown in Figure 12d.

However, in the case of backward inclined damage, due to the cubic topology optimization elements used in this study, they cannot be perfectly matched with the topology optimization elements, as shown in Figure 12e. Here, the cyan represents the healthy region compared to the predicted model, while the magenta indicates the damaged area. As a result, the model reconstruction may exhibit a sawtooth-like phenomenon, leading to some errors in the estimation of the damaged area. Nevertheless, it still maintains a considerable level of accuracy.

Parameter scanning is conducted on the reconstruction results of the structurally damaged area and the healthy area to establish the final updated parameters. The discrepancies between updated and default parameters are calculated and summarized in Table 4.

## 5. Model Updating for Damage Detection in Actual Experiments

### 5.1. Actual Experiments Initial Parameter Assessment

**Figure 13:**
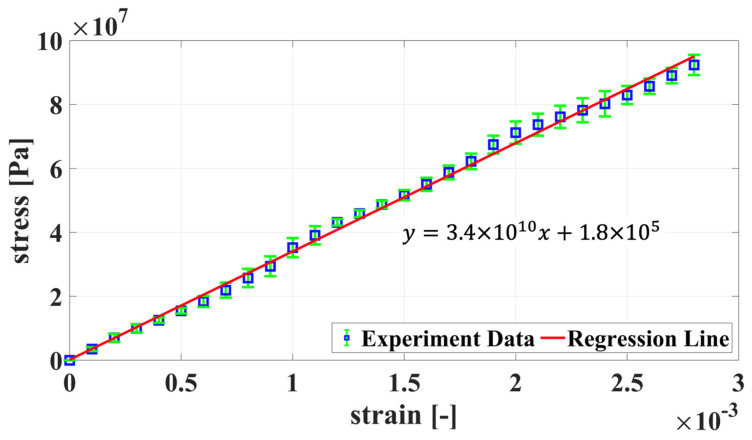
Initial parameter assessment: stress–strain curve.

The material used in the actual experiments of this study is a composite material. The composite laminate consists of T800 glass fiber and an epoxy resin matrix. It comprises eight layers of cross-ply glass fiber-reinforced polymer laminate, with a sequence of [0/90]_2S_. To establish the initial model, we first conducted tests on healthy, undamaged material specimens using a tensile testing machine to assess the basic parameters of the specimens used. Stress–strain curves were plotted based on measured strain and the stress from the tensile testing machine, as shown in Figure 13. A total of five sets of tests were conducted, and the data obtained from each test were consolidated and plotted to show the average stress–strain curve and its error range. The regression line slope was then taken as Young’s modulus, which was determined to be 34 GPa.

### 5.2. Model Updating by Stereo DIC

#### 5.2.1. Experimental Objectives and Experiment Case Setup

**Figure 14:**
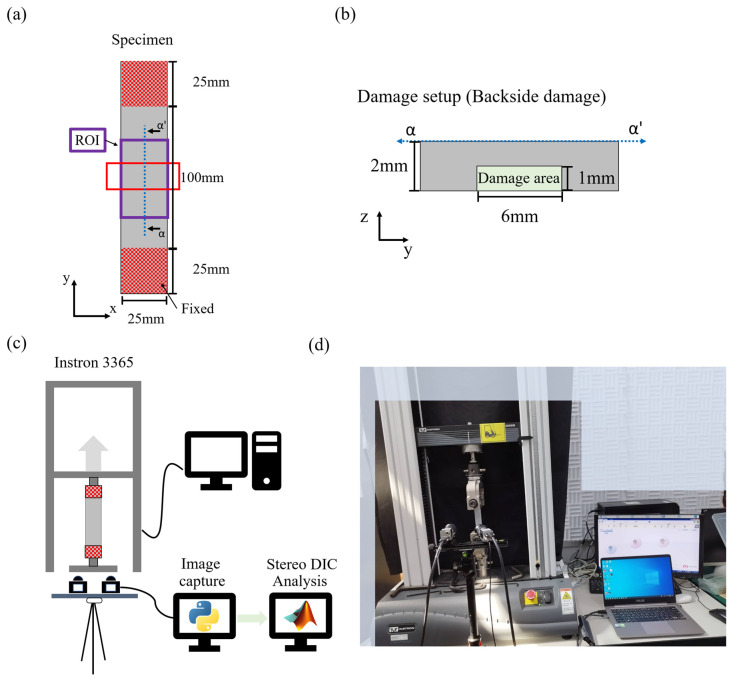
Actual experiment setup: (**a**) experiment specimen setup and (**b**) damage; experiment setup (**c**) schematic diagram and (**d**) actual experimental situation.

To validate the model updating process established in this study alongside the actual surface inspection data obtained through Stereo DIC, real experimental data will be used. Unlike numerical experiments under ideal conditions, real experimental data include noise, with displacement errors around 10^−3^ mm and strain errors around 10^−4^. This validation aims to confirm that the proposed FEMU method can be applied to experimental data with noise obtained through imaging techniques, specifically for detecting and assessing hidden damages. Therefore, in this section, actual damaged specimens will be fabricated, specifically focusing on setting up back damage scenarios as illustrated in Figure 14a,b. In the actual damage scenario, a groove was made to remove part of the structure, simulating the occurrence of damage. These specimens will undergo deformation through tensile testing using a universal testing machine. The surface changes will be analyzed using Stereo DIC to provide experimental data for the FEMU process. Based on the actual experimental results, FEMU process will be conducted to obtain reconstructed results, facilitating damage detection and assessment.

#### 5.2.2. Experiment Setup and Stereo DIC Results

In the actual experiment, tension will be applied to the specimens using a tensile testing machine to induce displacement. Images will be captured using a dual-camera system, as shown in Figure 14c,d. Python will be used to control both cameras and capture images simultaneously for synchronization.

**Figure 15:**
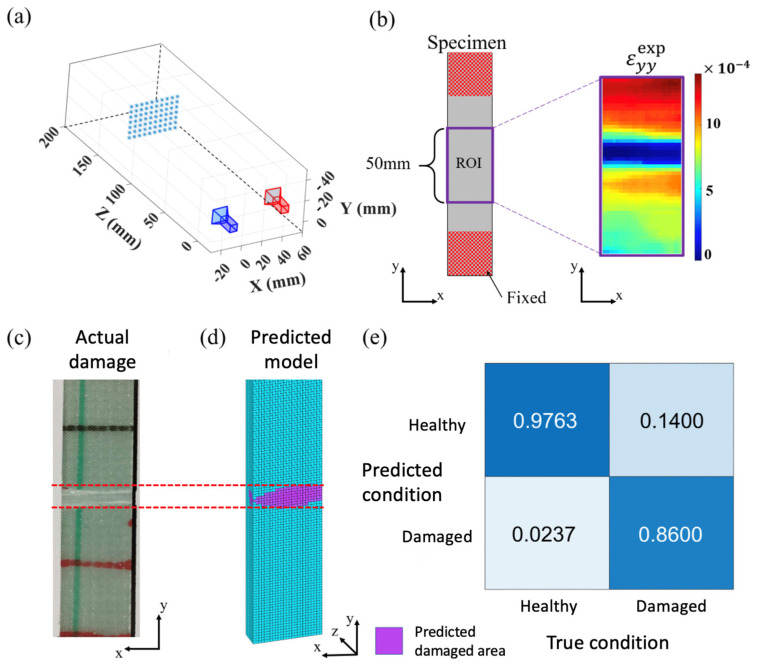
Results of actual experiment damage detection: (**a**) camera calibration; (**b**) stereo DIC detection results; (**c**) actual damaged specimens; (**d**) predicted damaged specimens; (**e**) accuracy analysis results.

Next, the analysis will be conducted using DUO DIC, which is based on MATLAB, to perform stereo DIC [35]. In the execution of stereo DIC, the camera pose will first be determined by capturing images of the calibration board, as shown in Figure 15a. Subsequently, similarity matching will be performed, and based on the results of camera pose estimation and similarity matching, calculations and analysis will be conducted to obtain the analysis results within the ROI, as depicted in Figure 15b. These analysis results represent the strain field in the Y direction. In the detection results, it can be observed that there is a region with relatively small numerical values in the middle. This region corresponds to the phenomenon of compression on the surface due to the influence of damage in the back area. The upper and lower parts of this region should exhibit a uniform distribution in finite element analysis, but at this moment, there is a noticeable progressive numerical variation. The reason for this difference may be attributed to the presence of more variables in the actual experiment compared to the ideal conditions in the simulation environment. Among these variables, the most likely reason is the slight discrepancy between the actual boundary conditions during tension and the simulated conditions.

#### 5.2.3. Actual Experiment Damage Detection Results

In the FEMU process, due to the surface information obtained during the actual experiments being limited to the ROI, this study only analyzes and calculates the model updates for that specific area, assuming the remaining parts as healthy regions. Under this circumstance, the two-stage model updating process proposed in this research is employed to update the structural numerical model using the actual experimental results. The feasibility has been experimentally verified of using surface-wide static information obtained through image methods for model updating, rather than solely discussing the ideal conditions under simulation scenarios. In addition, the reconstruction results can detect and assess damage beneath the surface, as shown in Figure 15c,d. Figure 15c depicts the actual fabricated structural damage model, while Figure 15d shows the predicted model computed using the proposed method. In these figures, cyan indicates the healthy region, while magenta represents the damaged area.

It is observed that due to discrepancies between experimental results obtained and the ideal conditions simulated, the reconstructed results exhibit some differences. For instance, the surface strain field of the experimental data may deviate from the numerical simulation results due to experimental conditions or errors during the actual damage fabrication process. These differences are reflected in the reconstructed model, leading to slight inaccuracies in estimating the damaged areas, consequently resulting in a decrease in the effectiveness of both damaged area estimation and parameter estimation. However, there is still a considerable level of accuracy in estimating the location of damaged areas, as shown in Figure 15e, achieving up to 86%.

## 6. Conclusions

The focus of this study lies in utilizing non-contact imaging methods for damage detection and assessment, aiming to establish an effective detection and analysis approach to restore the true structural condition. It addresses the detection of internal damages that cannot be directly identified through surface image analysis. Validation and discussion of the proposed method are conducted through numerical experiments under boundary conditions where the structure is subjected to tension, followed by practical experiments aimed at detecting concealed damage. The proposed method combines Stereo DIC with FEMU techniques for structural damage detection and analysis. Initially, Stereo DIC is utilized to obtain full-field displacement and strain information on the structure’s surface. Concurrently, a finite element analysis model is established to closely resemble the actual conditions. Subsequently, the finite element model is adjusted through a model updating process to align the analysis results with experimental data. This process involves structural reconstruction and parameter assessment, allowing for the detection and evaluation of internal damages within the structure. The research conclusions and recommendations for future research directions are summarized as follows:

1. The proposed approach integrates surface data obtained through image methods with FEMU techniques to detect and evaluate hidden damages beneath the surface, overcoming the limitations of image methods. Although this may require substantial computation time and resources—in our study, using a CPU 12th Gen Intel(R) Core (TM) i7-12700K required 3 days to 1 week of computation—this method overcomes the limitations of traditional imaging techniques, which is the main advantage of the proposed method. How to improve the analysis process and increase computational efficiency is a direction for future research and discussion.
2. The establishment of a dual-camera system in practical experiments enabled the acquisition of surface data using Stereo DIC. While there were discrepancies between the obtained detection results and the finite element analysis results, utilizing the method proposed in this study facilitated model reconstruction. This allowed for the detection and assessment of damage beneath the surface, with an estimated accuracy of approximately 86% for damaged area estimation. Thus, this validates the feasibility of integrating the results obtained from image analysis into the proposed method.
3. The methodological framework proposed in this study is preliminary; thus, it has primarily been tested and validated under ideal conditions (i.e., numerical experiments) and actual tensile tests. In the future, as this technology undergoes further development, more complex experimental boundary conditions, structural damage, material characteristics, and computational efficiency will be considered for more in-depth discussion.

## Figures and Tables

**Table 1:** Damage model parameter setting.

E_0_ = 74 [GPa]	Case 1 E_H_	Case 1 E_D_	Case 2 E_H_	Case 2 E_D_	Case 3 E_H_	Case 3 E_D_
Young’s modulus	74 [GPa]	4.7 [GPa]	74 [GPa]	4.7 [GPa]	50 [GPa]	4.7 [GPa]

**Table 2:** Parameter reconstruction table.

Case		Default Parameters	Step #1 Parameter	Step #1 Error	Final Parameter	Error
Case 1	E_H_	74 [GPa]	74 [GPa]	0%	67 [GPa]	10%
	E_D_	4.7 [GPa]	10.8 [GPa]	130%	3.1 [GPa]	34%
Case 2	E_H_	74 [GPa]	74 [GPa]	0%	67 [GPa]	10%
	E_D_	4.7 [GPa]	9.9 [GPa]	110%	5.5 [GPa]	17%
Case 3	E_H_	50 [GPa]	74 [GPa]	50%	53 [GPa]	7.8%
	E_D_	4.7 [GPa]	11 [GPa]	134%	4.1 [GPa]	13%

**Table 3:** Parameter error discussion settings.

Group	1	2	3	4	5
E_H_ [GPa]	37	50	74	110	150
E_H_/E_0_	1/2	2/3	1	3/2	2

**Table 4:** Damage case discussion settings and reconstruction.

Case	E_0_ = 74 [GPa]	Default Parameters	Final Parameter	Error
Case 4	E_H_	74 [GPa]	60 [GPa]	20%
	E_D_	4.7 [GPa]	3.7 [GPa]	21%
Case 5	E_H_	56 [GPa]	60 [GPa]	7%
	E_D_	4.7 [GPa]	3.7 [GPa]	21%
Case 6	E_H_	100 [GPa]	90 [GPa]	10%
	E_D_	4.7 [GPa]	6.1 [GPa]	29%

## Data Availability

The data presented in this study are available on request from the corresponding author.
